# P-1732. Evaluating the Impact of a Gram-Negative Bacteremia Treatment Guideline Combined with Antimicrobial Stewardship Pharmacist Intervention

**DOI:** 10.1093/ofid/ofae631.1896

**Published:** 2025-01-29

**Authors:** Alex Peterson-Weber, Kendall Donohoe, Ryan W Chapin, Hawra J Al Lawati, Matthew S Lee, Christopher McCoy

**Affiliations:** Beth Israel Deaconess Medical Center, Norfolk, Virginia; Beth Israel Deaconess Medical Center, Norfolk, Virginia; Beth Israel Deaconess Medical Center, Norfolk, Virginia; Beth Israel Deaconess Medical Center, Norfolk, Virginia; Beth Israel Deaconess Medical Center, Norfolk, Virginia; Beth Israel Deaconess Medical Center, Norfolk, Virginia

## Abstract

**Background:**

Gram-negative rod (GNR) bloodstream infections (BSIs) in hospitalized patients are associated with significant morbidity and mortality if not treated appropriately. By contrast, use of overly broad antibiotics or long durations of therapy for GNR BSIs may increase risk for antimicrobial resistance and *C. difficile* infection. The purpose of this study was to evaluate outcomes of GNR BSI treatment before and after implementation of an institutional practice guideline paired with real-time antimicrobial stewardship (AS) review.

**Figure:**
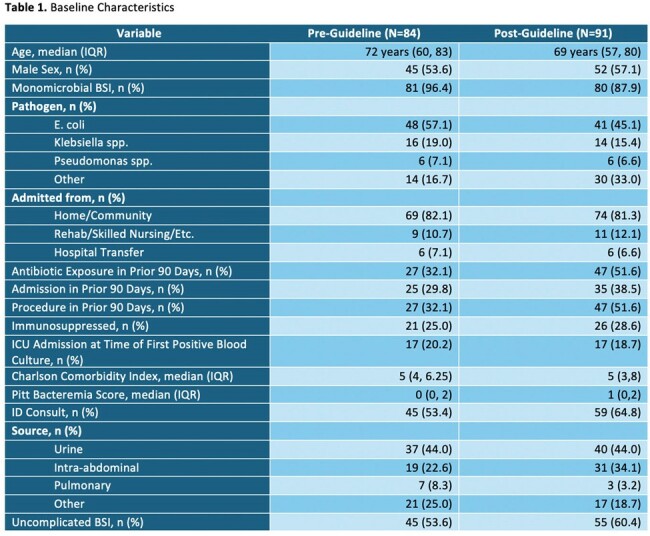

**Methods:**

A before-and-after quasi-experimental study was performed at our large academic medical center. Patients were identified for inclusion using a third-party pharmacovigilance platform based on presence of at least one blood culture with GNRs. Exclusion criteria included age less than 18 years, identified source of the GNR BSI was an infection requiring a prolonged duration of therapy (e.g., osteomyelitis, endovascular infection, etc.), or if the patient expired or was placed on comfort measures prior to BSI treatment completion. The primary endpoint was time to directed therapy, defined as susceptibility-directed tailoring of therapy. Secondary endpoints included time to optimal therapy (TOT), defined as narrowest effective therapy or step down to oral therapy, total duration of therapy, rate of step down to oral therapy, and acceptance of AS interventions.

**Figure:**
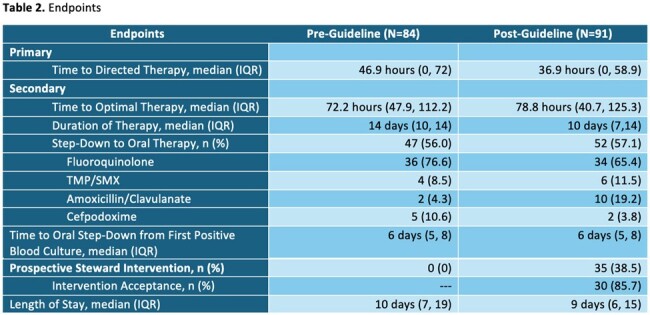

**Results:**

A total of 175 patients met inclusion criteria between November 2022 and April 2024. Eighty-four patients in the pre-guideline group and 91 patients in the post-guideline with AS review group were included. Baseline characteristics are described in table 1. The median time to directed therapy was 46.9 hours (IQR; 0, 72) in the pre-guideline group and 36.9 hours (IQR; 0, 58.9) in the post-guideline group. Additional endpoints are described in table 2.

**Conclusion:**

The GNR BSI guideline with AS review led to faster tailoring of therapy but no change in TOT. A high prevalence of Infectious Diseases consults was observed in this population, thus AS interventions were mostly directed at patients without formal Infectious Diseases consultation. Additionally, ability to make timely AS interventions was limited by the resources available to our AS program.

**Disclosures:**

**All Authors**: No reported disclosures

